# Assessment of Liver Fibrosis Using the Coefficient of Variation on Gadoxetic Acid-Enhanced Hepatobiliary Phase MR Imaging: A Prospective Multicenter Study

**DOI:** 10.3390/jcm15207996

**Published:** 2026-10-15

**Authors:** Youe Ree Kim, Tae-Hoon Kim, Chang-Won Jeong, Young Hwan Lee, Eun Young Cho, Keum Ha Choi, Yong Yeon Jeong, Suk Hee Heo, Kyung Won Kim, Yousun Ko, Youngbin Shin, Dong Min Kang, Kwon-Ha Yoon

**Affiliations:** 1 Department of Radiology, Wonkwang University Hospital, 895 Muwang-ro, Iksan 54538, Jeollabuk-do, Republic of Korea; 2 Medical Convergence Research Center, Wonkwang University, 460 Iksandae-ro, Iksan 54538, Jeollabuk-do, Republic of Korea; 3 Department of Internal Medicine, Wonkwang University Hospital, 895 Muwang-ro, Iksan 54538, Jeollabuk-do, Republic of Korea; 4 Department of Pathology, Wonkwang University Hospital, 895 Muwang-ro, Iksan 54538, Jeollabuk-do, Republic of Korea; 5 Department of Radiology, Chonnam National University Hwasun Hospital, 322 Seoyang-ro, Hwasun 58128, Jeollanam-do, Republic of Korea; 6 Department of Radiology, Asan Medical Center, 88 Olympic-ro 43-gil, Seoul 05505, Republic of Korea; 7 Biomedical Research Center, Asan Institute for Life Sciences, Asan Medical Center, 88 Olympic-ro 43-gil, Seoul 05505, Republic of Korea

**Keywords:** liver fibrosis, coefficient of variation, hepatobiliary phase, gadoxetic acid, quantitative MRI, METAVIR, non-invasive biomarker

## Abstract

**Background:** Accurate non-invasive assessment of liver fibrosis is needed in patients with chronic liver disease. Gadoxetic acid (Gd-EOB-DTPA)-enhanced hepatobiliary phase (HBP) magnetic resonance (MR) imaging reflects hepatocyte function and parenchymal heterogeneity, and the coefficient of variation (CV), which quantifies pixel-level signal inhomogeneity, has been proposed as a quantitative imaging biomarker of liver fibrosis. However, previous studies were retrospective, single-center, and lacked direct pathological validation. **Objectives:** To investigate the factors associated with CV on HBP images, evaluate the correlation between CV and pathologically confirmed liver fibrosis stage, and compare the diagnostic performance of CV with serum biomarkers, including the aspartate aminotransferase-to-platelet ratio index (APRI) and fibrosis-4 (FIB-4), in a prospective multicenter setting. **Methods:** This prospective multicenter study was conducted between April 2017 and August 2020 at three medical centers. Ninety-nine participants who underwent Gd-EOB-DTPA-enhanced HBP T1-weighted MRI and liver biopsy or surgery were enrolled. CV was measured on HBP images, and the METAVIR fibrosis stage was used as the reference standard. Univariate and multivariable linear regression analyses, including an additional model adjusted for institution and pathology confirmation method, Welch’s analysis of variance with the Games–Howell post hoc test, Spearman’s rank correlation analysis, receiver operating characteristic (ROC) curve analysis with DeLong’s method for AUROC comparison, and intraclass correlation coefficient (ICC) analyses were performed. **Results:** Ninety-nine participants (64 men and 35 women; mean age, 55.5 ± 15.1 years) were enrolled. In multivariable linear regression analysis adjusted for institution and pathology confirmation method, pathologic fibrosis stage (B = 0.468; 95% CI, 0.358–0.578; *p* < 0.001) and serum albumin level (B = −0.341; *p* = 0.015) were independently associated with CV. Spearman’s correlation coefficient between CV and fibrosis stage was 0.74 (*p* < 0.001). The AUROCs of CV for significant fibrosis (≥F2), advanced fibrosis (≥F3), and cirrhosis (F4) were 0.92, 0.88, and 0.86, respectively (all *p* < 0.001). CV outperformed APRI and FIB-4 across most fibrosis thresholds (*p* < 0.001 to *p* = 0.045), except for FIB-4 in the diagnosis of ≥F2 fibrosis (*p* = 0.09). The intra- and inter-observer ICC values were 0.94 and 0.84, respectively. **Conclusions:** In this prospective multicenter study with pathologically confirmed fibrosis stages, CV derived from Gd-EOB-DTPA-enhanced HBP MR images showed a strong correlation with liver fibrosis stage and higher diagnostic performance than APRI and FIB-4 across most fibrosis thresholds. Because CV can be calculated from routine clinical MRI acquisitions without additional hardware or blood tests, it may serve as a practical biomarker for non-invasive liver fibrosis assessment. However, because this study was conducted in a Korean cohort with predominantly hepatitis B virus-related liver disease, further validation in other ethnic groups and etiologies is needed.

## 1. Introduction

Evaluation of liver fibrosis in patients with chronic liver disease has important implications for clinical decision-making, including antiviral treatment, surveillance, and prevention of disease progression to cirrhosis and hepatocellular carcinoma (HCC) [1,2,3]. Although liver biopsy remains the reference standard for assessing liver fibrosis, it is invasive, costly, and limited by sampling error [4]. Therefore, various non-invasive methods have been developed as alternatives, including serum biomarkers and imaging modalities [2,5,6]. The aspartate aminotransferase-to-platelet ratio index (APRI) and fibrosis-4 (FIB-4) are indirect serum biomarkers based on liver function tests and clinical parameters associated with liver fibrosis [2,7,8,9]. In contrast, elastography using ultrasound or MRI allows direct measurement of tissue stiffness or shear wave propagation, whereas quantitative imaging approaches provide indirect analytical assessment [5,6,10,11,12]. Texture analysis refers to histogram-based analysis or quantification of liver parenchymal features derived from medical images [13,14,15,16].

Recent advances in quantitative MRI have expanded the non-invasive options available for liver fibrosis assessment. The 2024 American Association for the Study of Liver Diseases (AASLD) Practice Guideline on imaging-based noninvasive liver disease assessment endorsed the use of imaging-based modalities for staging significant fibrosis (≥F2), advanced fibrosis (F3–F4), and cirrhosis (F4), and noted that imaging-based approaches generally outperform blood-based biomarkers in diagnostic accuracy [17]. Nevertheless, access to specialized hardware such as MR elastography remains limited in many clinical settings; therefore, quantitative methods that can be derived from routine MRI acquisitions are needed. Gadoxetic acid-enhanced MRI has become a preferred modality for liver MRI because it provides both morphologic and functional information within a single examination [18].

Gadolinium ethoxybenzyl-diethylenetriaminepentaacetic acid (Gd-EOB-DTPA; gadoxetic acid, Primovist^®^, Bayer Healthcare, Berlin, Germany) is a liver-specific MRI contrast agent taken up into hepatocytes via organic anion-transporting polypeptides (OATP1B1/1B3) and excreted through the hepatobiliary system, enabling generation of hepatocyte-specific hepatobiliary phase (HBP) images 15–20 min after injection. Signal intensity on HBP images reflects both hepatic function and the degree of liver fibrosis [13,19,20,21]. The heterogeneity of hepatic parenchymal pixels on HBP images can be expressed as the coefficient of variation (CV), defined as the standard deviation of pixel signal intensities within a region of interest divided by their mean, multiplied by 100 and expressed as a percentage, which quantifies the degree of signal heterogeneity in the liver parenchyma. CV measurement on HBP images has been proposed as a quantitative imaging method for liver fibrosis assessment [13,22,23]. However, previous studies were retrospective, conducted at a single institution, and relied on indirect comparisons between CV and serum fibrosis biomarkers such as APRI and FIB-4 rather than direct pathological validation [22,23]. In addition, factors other than fibrosis that may influence CV have not been fully investigated.

Therefore, this prospective multicenter study was designed to evaluate the relationship between CV measured on Gd-EOB-DTPA-enhanced HBP MRI and pathologically proven liver fibrosis. The purpose of this study was to investigate the factors associated with CV, assess the relationship between CV and histopathologic fibrosis stage, and compare the diagnostic performance of CV with APRI and FIB-4 in patients with pathologically confirmed liver fibrosis.

## 2. Materials and Methods

### 2.1. Study Design

This prospective multicenter study was conducted at three medical centers from April 2017 through August 2020. Institution A was Wonkwang University Hospital (Iksan, Jeollabuk-do), Institution B was Chonnam National University Hwasun Hospital (Hwasun, Jeollanam-do), and Institution C was Asan Medical Center (Seoul). The study was approved by the institutional review board of Institution A (IRB No. 201607-CTDG-016), as well as by the institutional review boards of the other participating centers. Because the METAVIR fibrosis staging system comprises five stages, we planned to enroll approximately 100 participants, targeting at least 15–20 participants per fibrosis stage. We also aimed to include similar numbers of participants undergoing surgery and biopsy, as well as fewer than 15 healthy controls. After review of the clinical data according to the inclusion and exclusion criteria at each institution, anonymized MRI, clinical, and pathological data were collected and centralized at Institution A.

### 2.2. Participant Selection

**Figure 1:**
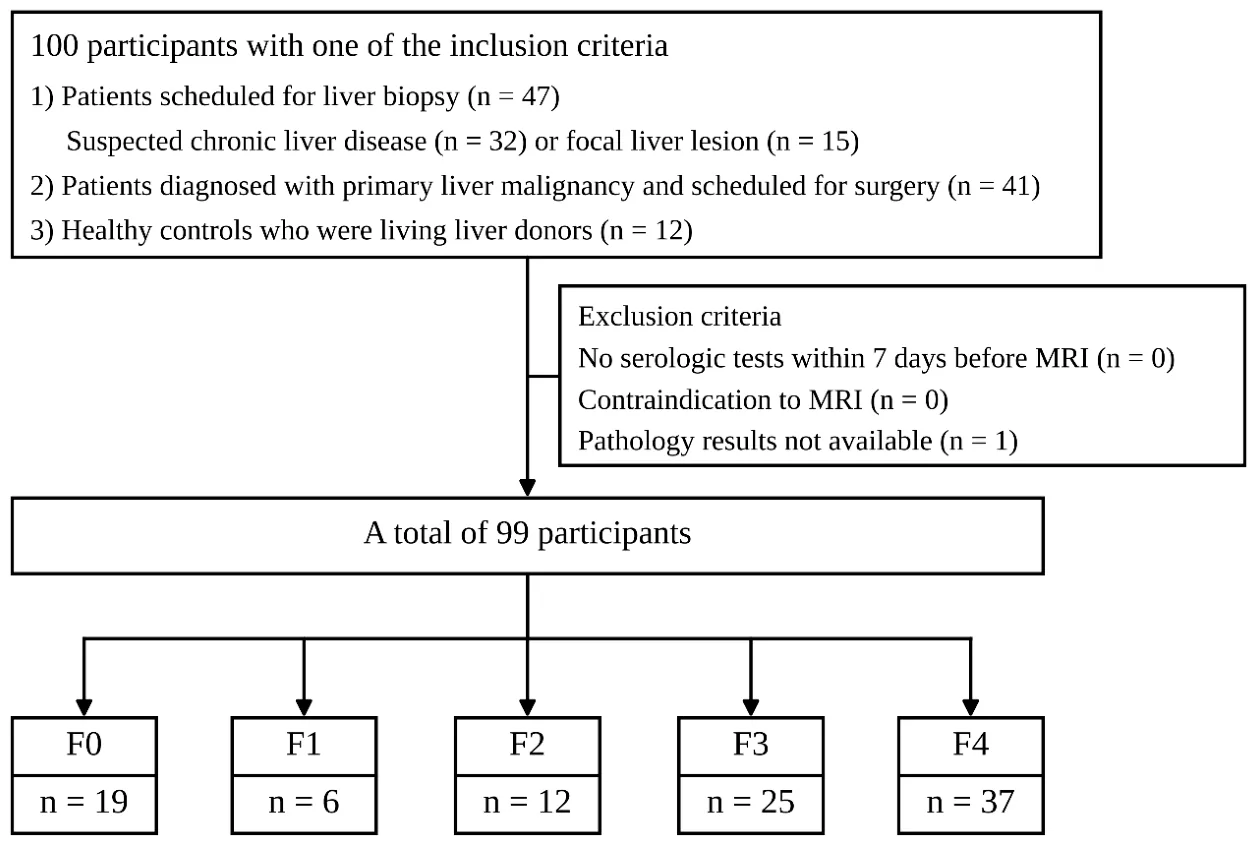
Flow diagram of participant selection. METAVIR fibrosis stages: F0, no fibrosis; F1, portal fibrosis without septa; F2, portal fibrosis with few septa; F3, numerous septa without cirrhosis; F4, cirrhosis.

All participants provided written informed consent before enrollment. The study included participants who met one of the following inclusion criteria: (1) patients scheduled for liver biopsy (*n* = 47) because of suspected chronic liver disease (*n* = 32) or a focal liver lesion (*n* = 15); (2) patients diagnosed with primary liver malignancy and scheduled to undergo surgery (*n* = 41); and (3) healthy controls who were living liver donors (*n* = 12). Participants were excluded if they met one of the following criteria: (1) absence of serologic tests performed within 7 days before MRI (*n* = 0); (2) contraindication to MRI, including claustrophobia, estimated glomerular filtration rate < 30 mL/min/1.73 m^2^, or adverse reactions to contrast material (*n* = 0); or (3) unavailable pathological results because neither surgery nor biopsy had been performed (*n* = 1). A total of 100 participants were initially enrolled; one participant was excluded according to the exclusion criteria. Thus, the final study population comprised 99 participants (Figure 1).

### 2.3. Imaging Acquisition

The detailed MRI protocols for each institution are provided in the Supplementary Materials. In brief, MRI was performed using 3-T scanners across the participating institutions. Hepatobiliary phase images were acquired using a three-dimensional fat-suppressed T1-weighted sequence at 15 or 20 min after intravenous administration of Gd-EOB-DTPA (Primovist; Bayer Healthcare, Berlin, Germany).

### 2.4. CV Analysis

After MRI acquisition, all HBP images were collected at Institution A in the Digital Imaging and Communications in Medicine (DICOM) format. CV values were measured by one abdominal radiologist (Y.R.K.) with 10 years of experience in abdominal imaging, blinded to the participants’ clinical and pathological information. CV maps were generated using custom-developed software written in MATLAB (R2018a; MathWorks, Natick, MA, USA). After image loading, bias-field correction and semi-automatic liver segmentation were performed using images obtained at the level of the hepatic hilum [22]. Details of the bias-field correction procedure are provided in the Supplementary Materials. Three fixed-size circular regions of interest (ROIs), each comprising more than 40 pixels (approximately 150–200 mm^2^), were placed in liver segment IV (left medial segment), the right anterior section (segments V and VIII), and the right posterior section (segments VI and VII), while avoiding visible bile ducts, vessels, focal lesions, and the subcapsular area. The left lateral segment (segments II and III) was excluded from the outset because artifacts were frequently present in this region. No separate formal reader training or calibration session was performed. The software displayed overlays identifying vessels and focal lesions, which readers verified before manually placing the circular ROIs. Within each anatomical region, exclusion of vessels, focal lesions, and the subcapsular area restricted the parenchymal area available for the fixed-size ROI. As a result, the range of eligible ROI locations was limited. Each ROI was measured three times, and the mean CV value per ROI was used for analysis. The final CV value for each participant was calculated as the mean of the three regional measurements. The equations used for CV mapping are provided in the Supplementary Materials.

To assess intra-observer reliability, repeated measurements were performed by the same reader (Y.R.K.) after 4 weeks. Two additional abdominal radiologists (D.M.K. and K.H.Y.) with 11 and 21 years of experience in abdominal imaging, respectively, independently analyzed the CV maps blinded to each other’s measurements and to clinical and pathological information, for assessment of inter-observer agreement.

### 2.5. Laboratory Tests and Serum Biomarkers

Platelet count and serum levels of bilirubin and albumin, which are associated with liver fibrosis and cirrhosis, were collected. Liver enzyme levels, including aspartate aminotransferase (AST), alanine aminotransferase (ALT), alkaline phosphatase (ALP), and gamma-glutamyltransferase (GGT), were also measured. Blood samples were analyzed at the clinical laboratory of each respective institution using standard laboratory protocols. Viral markers associated with chronic hepatitis B or C, including hepatitis B surface antigen (HBsAg), hepatitis B surface antibody (HBsAb), and hepatitis C virus antibody (anti-HCVAb), were recorded. The serum fibrosis biomarkers APRI and FIB-4 were calculated as previously described [8].

### 2.6. Pathologic Fibrosis Staging

**Figure 2:**
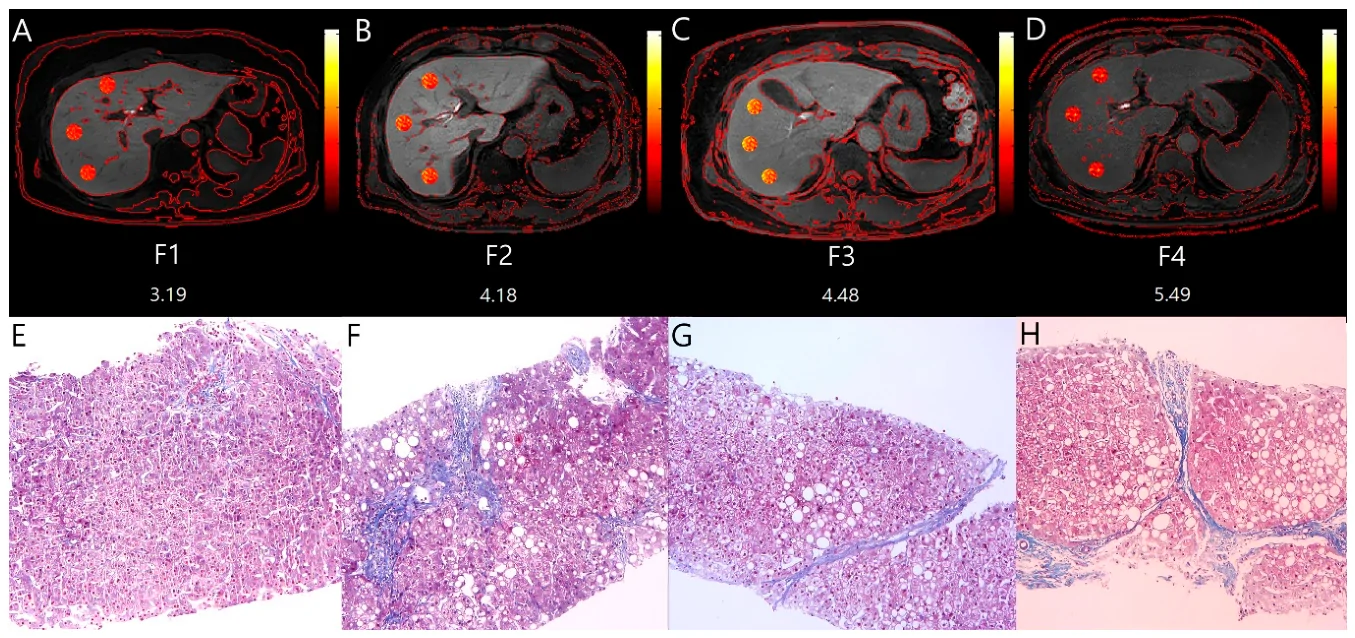
Representative cases of CV measurements on hepatobiliary phase MR images and corresponding histopathologic findings (Masson’s trichrome stain, ×10) according to METAVIR fibrosis stage. (**A**) A representative patient with a CV value of 3.19 and portal fibrosis without septa (F1). (**B**) A representative patient with a CV value of 4.18 and portal fibrosis with few septa (F2). (**C**) A representative patient with a CV value of 4.48 and numerous septa without cirrhosis (F3). (**D**) A representative patient with a CV value of 5.49 and cirrhosis (F4). (**E**–**H**) Corresponding Masson’s trichrome-stained sections from the patients shown in (**A**–**D**), respectively. Collagen fibers are stained blue.

Pathologic specimens were reviewed by one dedicated hepatopathologist at each institution (three pathologists in total), each blinded to clinical and imaging findings. Liver fibrosis was staged according to the METAVIR scoring system as follows: F0, no fibrosis; F1, portal fibrosis without septa; F2, portal fibrosis with few septa; F3, numerous septa without cirrhosis; and F4, cirrhosis [24]. Representative formalin-fixed, paraffin-embedded liver tissue sections (4 µm) were stained with Masson’s trichrome to visualize collagen deposition corresponding to each METAVIR fibrosis stage, and representative images from each stage (F1–F4) are shown in Figure 2.

### 2.7. Statistical Analysis

CV values, serum biomarkers, and laboratory test results are presented as mean ± standard deviation. Baseline characteristics were compared between the F0 and F1–F4 groups using the Mann–Whitney U test for continuous variables and the chi-square test or Fisher’s exact test for categorical variables. Normality within each fibrosis stage was assessed using the Shapiro–Wilk test, and homogeneity of variance was assessed using Levene’s test. Because the assumption of equal variances was not met, mean CV values across fibrosis stages (F0–F4) were compared using Welch’s analysis of variance (ANOVA), with the Games–Howell test applied for post hoc pairwise comparisons. Univariate and multivariable linear regression analyses were initially performed to identify clinical and laboratory factors associated with CV. In addition, a separate adjusted multivariable model including institution and pathology confirmation method was constructed to assess whether the association between fibrosis stage and CV remained robust after accounting for clinical-pathway and center-related heterogeneity. Pathology confirmation method was coded as 0 for surgery and 1 for biopsy, and collinearity was assessed using variance inflation factors (VIFs). Because the METAVIR fibrosis stage is an ordinal variable, the relationship between CV and fibrosis stage was evaluated using Spearman’s rank correlation coefficient. The diagnostic performance of mean CV, APRI, and FIB-4 for fibrosis staging was assessed using receiver operating characteristic (ROC) curve analysis, with optimal cutoff values determined using the Youden index; AUROCs were compared using DeLong’s method. A sensitivity analysis restricted to surgically confirmed cases was also performed. Matched comparisons between groups stratified by HBP acquisition time (15 vs. 20 min) and across different MRI systems were performed using paired *t*-tests. Reliability was evaluated using intraclass correlation coefficients (ICCs) for intra- and inter-observer agreement based on absolute agreement. No missing data were present for the primary outcome variable (CV) or for any covariates included in the regression models. A two-sided *p* value < 0.05 was considered statistically significant. All statistical analyses were performed using SPSS for Windows, version 29.0 (IBM Corp., Armonk, NY, USA), and MedCalc Statistical Software, version 20.015 (MedCalc Software, Ostend, Belgium).

## 3. Results

### 3.1. Participant Characteristics

**Figure 3:**
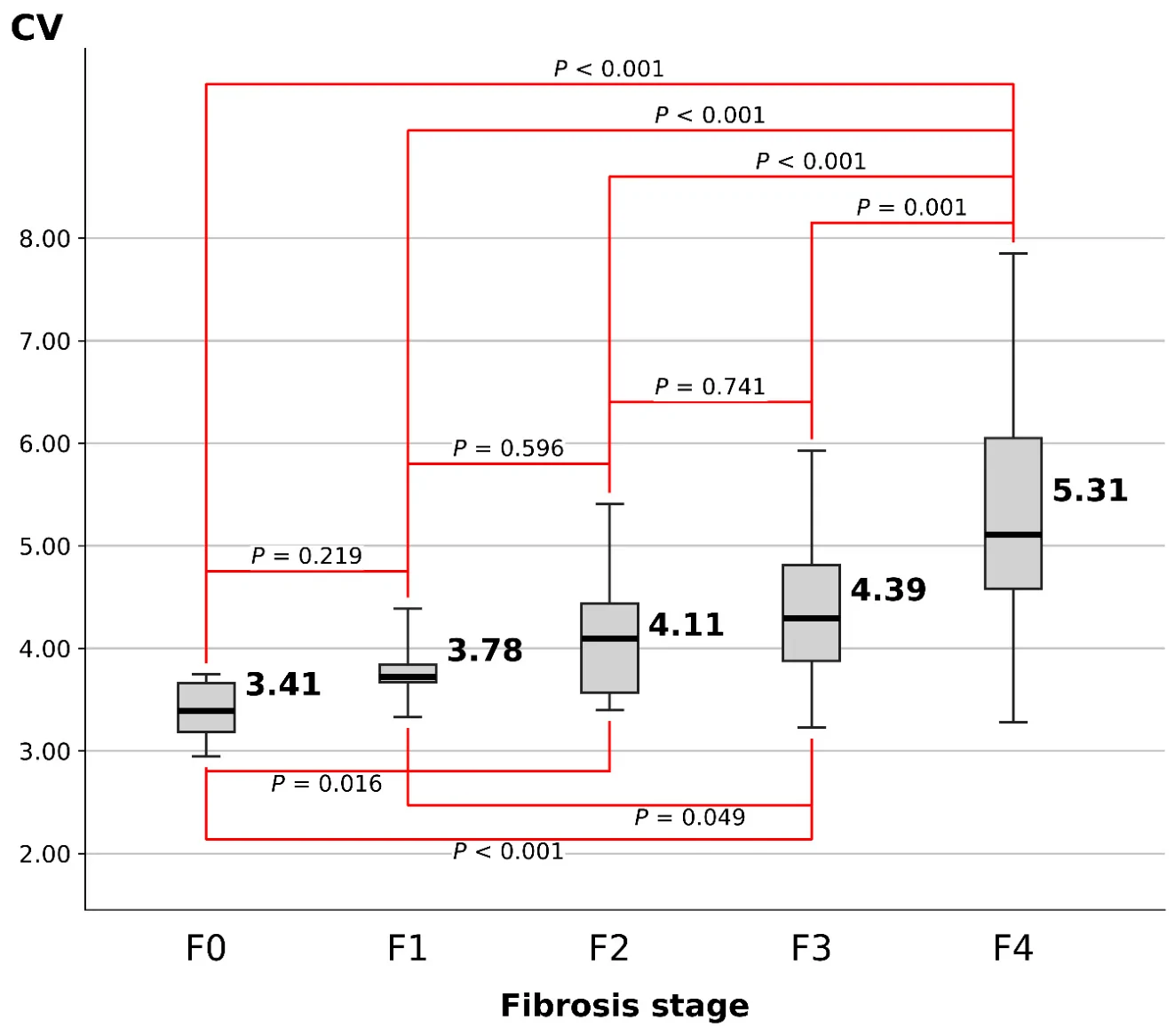
Box plots of the coefficient of variation (CV) according to METAVIR fibrosis stage. Gray boxes indicate the interquartile range, and red lines indicate pairwise comparisons between fibrosis stages, with corresponding *p* values obtained using the Games–Howell post hoc test. Numbers beside the boxes indicate mean CV values. CV correlated with fibrosis stage (Spearman’s ρ = 0.74, *p* < 0.001).

Of the 99 participants, 19 were pathologically confirmed as having no fibrosis (F0; 12 men and 7 women; mean age, 37.5 ± 17.5 years), and 80 had histologically confirmed liver fibrosis (F1–F4; 52 men and 28 women; mean age, 59.8 ± 10.8 years). Among the fibrosis patients, the most common etiologies were hepatitis B (*n* = 33), alcohol-related liver disease (*n* = 14), and autoimmune hepatitis (*n* = 12). The distribution of pathologic fibrosis stages was as follows: F0, 19 participants; F1, 6 participants; F2, 12 participants; F3, 25 participants; and F4, 37 participants (Figure 1 and Table 1). Mean CV values differed significantly according to METAVIR fibrosis stage (Welch’s ANOVA, *p* < 0.001): 3.41 ± 0.27 in F0, 3.78 ± 0.35 in F1, 4.11 ± 0.62 in F2, 4.39 ± 0.69 in F3, and 5.31 ± 1.06 in F4. In Games–Howell post hoc comparison, CV in F4 was higher than in all other stages (*p* ≤ 0.001), and CV in F0 was lower than in F2 (*p* = 0.016) and F3 (*p* < 0.001) (Figure 3). The mean values and standard deviations of the serologic test results are summarized in Table 1.

Numbers beside the boxes indicate mean CV values. *p* values were obtained using Welch’s ANOVA with the Games–Howell post hoc test. CV correlated with fibrosis stage (Spearman’s ρ = 0.74, *p* < 0.001).

### 3.2. Factors Associated with CV

Laboratory variables and pathologic fibrosis stage were analyzed to identify factors associated with CV (Table 2). In univariate linear regression analysis, platelet count, ALP, albumin level, and pathologic fibrosis stage were significantly associated with CV (all *p* < 0.001). In the initial clinical-factor multivariable model, serum albumin level and pathologic fibrosis stage remained significantly associated with CV (*p* = 0.047 and *p* < 0.001, respectively).

In a separate adjusted model accounting for institution and pathology confirmation method, fibrosis stage remained independently associated with CV (B = 0.468; 95% CI, 0.358–0.578; *p* < 0.001). Albumin was inversely associated with CV (B = −0.341; 95% CI, −0.615 to −0.067; *p* = 0.015), and biopsy, compared with surgery, was associated with a higher CV (B = 1.023; 95% CI, 0.452–1.593; *p* = 0.001) (Supplementary Table S1).

In the sensitivity analysis restricted to surgically confirmed cases, fibrosis stage remained significantly associated with CV (B = 0.381; 95% CI, 0.216–0.546; *p* < 0.001) (Supplementary Table S2).

### 3.3. Relationship Between CV and Fibrosis Stage

CV showed a strong positive correlation with fibrosis stage (Spearman’s ρ = 0.74, *p* < 0.001) (Figure 3).

### 3.4. Diagnostic Performance of CV, APRI, and FIB-4 for Liver Fibrosis Staging

**Figure 4:**
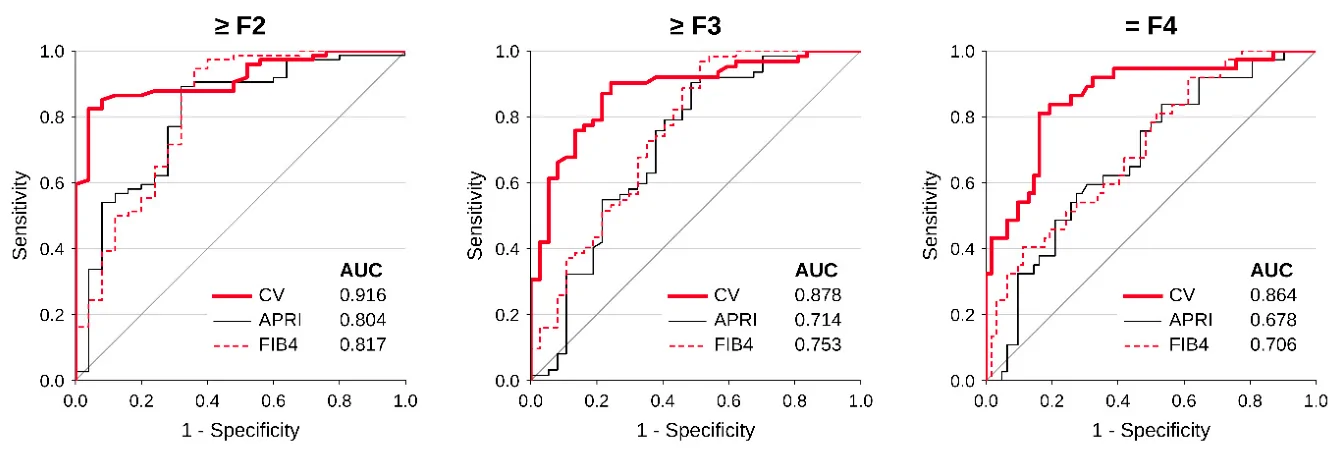
Receiver operating characteristic (ROC) curves of mean CV, APRI, and FIB-4 for the diagnosis of significant fibrosis (≥F2), advanced fibrosis (≥F3), and cirrhosis (F4). The AUROCs of CV were 0.92 for ≥F2, 0.88 for ≥F3, and 0.86 for F4 (all *p* < 0.001).

The AUROCs of mean CV, APRI, and FIB-4 for diagnosing liver fibrosis are summarized in Table 3 and Figure 4. The AUROCs of mean CV for diagnosing significant fibrosis (≥F2), advanced fibrosis (≥F3), and cirrhosis (F4) were 0.92 (95% CI, 0.86–0.97), 0.88 (95% CI, 0.81–0.95), and 0.86 (95% CI, 0.79–0.94), respectively (all *p* < 0.001). Using the Youden index, the optimal cutoff value of CV for significant fibrosis (≥F2) was 3.84, yielding a sensitivity of 82.4% and a specificity of 96.0%. For cirrhosis (F4), the optimal cutoff value was 4.50, yielding a sensitivity of 81.1% and a specificity of 83.9%.

The AUROCs of APRI were 0.80 (95% CI, 0.70–0.91; *p* < 0.001) for ≥F2, 0.71 (95% CI, 0.60–0.83; *p* < 0.001) for ≥F3, and 0.68 (95% CI, 0.57–0.79; *p* = 0.001) for F4. The corresponding AUROCs of FIB-4 were 0.82 (95% CI, 0.71–0.93; *p* < 0.001), 0.75 (95% CI, 0.65–0.86; *p* < 0.001), and 0.71 (95% CI, 0.60–0.81; *p* < 0.001), respectively.

### 3.5. Comparison of Diagnostic Performance Between CV, APRI, and FIB-4

The AUROCs of mean CV were higher than those of APRI and FIB-4 across most fibrosis thresholds. For significant fibrosis (≥F2), the AUROC of CV was higher than that of APRI (0.92 vs. 0.80, *p* = 0.045), whereas the difference between CV and FIB-4 did not reach statistical significance (0.92 vs. 0.82, *p* = 0.09). For advanced fibrosis (≥F3), CV outperformed APRI (0.88 vs. 0.71, *p* = 0.005) and FIB-4 (0.88 vs. 0.75, *p* = 0.03). For cirrhosis (F4), CV also showed significantly higher AUROCs than APRI (0.86 vs. 0.68, *p* < 0.001) and FIB-4 (0.86 vs. 0.71, *p* = 0.006).

### 3.6. Comparison According to HBP Acquisition Time and MRI System

To assess whether CV values were consistent across images acquired on different MRI systems, 15 participants from Institutions A and C were matched according to histologic stage and compared using a paired *t*-test. The mean CV value was 4.31 ± 1.16 in Institution A and 4.32 ± 1.06 in Institution C, with no significant difference (*p* = 0.99).

To compare images obtained at different HBP acquisition times, 20 participants from Institution B with 15-min HBP images were matched according to histologic stage with participants from Institutions A and C with 20-min HBP images, and the groups were compared using a paired *t*-test. The mean CV value was 4.28 ± 0.65 in Institution B and 4.56 ± 0.54 in Institutions A and C, without a statistically significant difference (*p* = 0.09).

### 3.7. Intra- and Inter-Reader Variability

For CV measurements, the intra-observer agreement of reader 1 was excellent, with an ICC of 0.94 (95% CI, 0.91–0.96; *p* < 0.001). The inter-observer agreement among the three readers was also good, with an ICC of 0.84 (95% CI, 0.75–0.86; *p* < 0.001).

## 4. Discussion

This prospective multicenter study evaluated the usefulness of coefficient of variation mapping for the assessment of liver fibrosis, using pathologic findings as the reference standard. In multivariable analysis, CV was significantly associated with pathologic fibrosis stage and serum albumin level, and both associations persisted after additional adjustment for institution and pathology confirmation method. In addition, CV showed a strong positive correlation with METAVIR fibrosis stage and demonstrated better diagnostic performance than the serum biomarkers APRI and FIB-4 across most fibrosis thresholds.

CV measurement is based on texture analysis of hepatic enhancement after administration of Gd-EOB-DTPA. Gadoxetic acid is taken up into hepatocytes mainly via OATP1B1 and OATP1B3 and excreted into bile via MRP2. In liver fibrosis, reduced hepatobiliary phase enhancement is thought to be driven primarily by decreased hepatocellular uptake associated with impaired OATP-mediated transport [25,26,27,28]. These changes, together with collagen deposition in the space of Disse during hepatic fibrosis, may impair contrast transport and contribute to heterogeneous distribution of contrast material within the liver parenchyma. This may increase parenchymal signal heterogeneity on HBP images.

The two previous studies investigating CV were performed without direct comparison with pathological findings and did not comprehensively evaluate factors related to fibrosis and inflammation that may influence signal heterogeneity on HBP images [22,23]. In the present study, multivariable analysis demonstrated that pathologic fibrosis stage (*p* < 0.001) and serum albumin level (*p* = 0.047; *p* = 0.015 in the adjusted model) were significantly associated with CV, with fibrosis stage showing the strongest association. Lee et al. [22] previously reported moderate correlations between CV and serum biomarkers such as APRI and FIB-4 (Spearman’s ρ = 0.39 and 0.29, respectively; both *p* < 0.001). In contrast, we found a strong positive correlation between CV and METAVIR fibrosis stage, with histopathology used as the reference standard (Spearman’s ρ = 0.74, *p* < 0.001).

Although serum biomarkers such as APRI and FIB-4 are widely used as non-invasive surrogates of liver fibrosis, they reflect indirect biochemical consequences of hepatic dysfunction rather than parenchymal changes themselves. CV derived from Gd-EOB-DTPA-enhanced HBP images quantifies pixel-level signal inhomogeneity within the hepatic parenchyma, which may increase as functioning hepatocytes are progressively replaced by fibrotic tissue. The strong correlation between CV and pathologically confirmed fibrosis stage in the present study may reflect this mechanism. Furthermore, unlike APRI and FIB-4, which require laboratory values that may fluctuate with inflammation, medication use, or other clinical conditions, CV can be derived from MRI datasets that are already acquired during routine clinical evaluation of liver disease without additional blood sampling or specialized hardware. This may be a practical advantage in patients who are already undergoing Gd-EOB-DTPA-enhanced MRI for lesion characterization or preoperative evaluation. Traditionally, indirect scoring systems such as APRI and FIB-4 have been used for non-invasive assessment of liver fibrosis [2,7,8,29]. In a previous meta-analysis, the pooled AUCs of APRI and FIB-4 for the diagnosis of hepatic fibrosis ranged from 0.74 to 0.84 in patients with chronic hepatitis B [8]. In the present study, the diagnostic performance of APRI and FIB-4 was comparable to that reported in previous studies, with AUROCs ranging from 0.68 to 0.82. However, CV showed significantly higher AUROCs than APRI and FIB-4 across most fibrosis thresholds, with *p* values ranging from <0.001 to 0.045, except for FIB-4 in the diagnosis of ≥F2 fibrosis (*p* = 0.09).

Several MRI-based methods have been proposed for non-invasive assessment of hepatic fibrosis, including analyses of liver morphology, contrast enhancement, signal intensity, and texture [5,6,12]. Morphometric approaches such as liver surface nodularity (LSN) quantify cirrhotic morphologic changes in the liver. In one study, MR-based LSN showed inferior performance compared with MR elastography for diagnosing significant fibrosis (AUROC, 0.61 vs. 0.87, respectively) [30]. Contrast-enhancement-based methods have also been investigated on the basis of the pharmacokinetics of hepatobiliary contrast agents [21,27,31,32]. In particular, contrast enhancement indices derived from MRI have shown AUROCs of 0.85 and 0.88 for significant fibrosis and cirrhosis, respectively [32]. Quantitative signal intensity and relaxation-time measurements have also been studied using T1-weighted imaging, T2-weighted imaging, diffusion-weighted imaging, and susceptibility-weighted imaging [33,34,35]. In addition, multiparametric approaches have been used for fibrosis assessment. For example, the liver-to-muscle ratio on susceptibility-weighted imaging showed a higher AUROC for diagnosing cirrhosis than relative enhancement and apparent diffusion coefficient values (0.92 vs. 0.80 vs. 0.79, respectively; *p* < 0.001), and the combination of these parameters yielded the highest AUROC for cirrhosis (0.93) [34]. More recently, a radiomics-based fibrosis index derived from gadoxetic acid-enhanced MRI demonstrated AUROCs of 0.90, 0.89, and 0.91 for significant fibrosis, advanced fibrosis, and cirrhosis, respectively [36]. In the present study, CV demonstrated good diagnostic performance, with AUROCs of 0.92 for ≥F2, 0.88 for ≥F3, and 0.86 for F4. Comparisons with performance metrics reported for other MRI-based methods are indirect because these methods were not evaluated in the same cohort. Such comparisons are hypothesis-generating only; direct head-to-head validation is required before conclusions regarding relative performance can be drawn. More recently, deep learning algorithms applied to gadoxetic acid-enhanced HBP MRI have been proposed for automated fibrosis staging, with AUROCs of 0.91–0.92 for significant fibrosis in test cohorts [37]. Deep learning approaches allow automated analysis but require large annotated training datasets and dedicated computational resources, and their generalizability across institutions and scanners has not been fully established. CV analysis may be a complementary option because it requires only standard DICOM images and a simple calculation. CV showed its highest AUROC for significant fibrosis (≥F2), a threshold commonly used for clinical decision-making. However, this estimate may have been influenced by the inclusion of healthy liver donors in the F0 group, and the difference from FIB-4 at this threshold was not statistically significant.

CV analysis has several practical advantages. First, liver fibrosis may be monitored using HBP images obtained during routine Gd-EOB-DTPA-enhanced liver MRI. Although MRI performed solely for CV analysis may not be cost-effective, patients with chronic liver disease often undergo repeated liver MRI for hepatocellular carcinoma surveillance, and CV could potentially be used to monitor fibrosis in this setting without repeated biopsy. Second, HBP images can be acquired using Gd-EOB-DTPA without the need for vendor-specific hardware or dedicated elastography equipment. In addition, CV can be calculated from standard DICOM images using relatively simple software, with bias-field correction and semi-automatic segmentation as the only preprocessing steps. Third, the measurement showed good reproducibility, with high intra- and inter-observer agreement (ICC, 0.94 and 0.84, respectively). Although ROI placement required reader input, the fixed ROI size and the limited eligible parenchymal area within each anatomical region constrained the choice of ROI location. These constraints may have contributed to the observed high intra-observer agreement and good inter-observer agreement, even without a separate reader training or calibration session. ROI placement was not fully automated, and some reader dependence remains. The inter-observer agreement in the present study was slightly lower than that reported in previous studies [22,23], possibly because three readers participated in this study and a different statistical approach was used.

This study has several limitations. First, the sample size was relatively small, and the number of participants across fibrosis stages was uneven, reflecting the constraints of a prospective study conducted in a real-world clinical setting. Enrollment through distinct clinical pathways, including biopsy, surgical resection, and living-donor evaluation, may have contributed to the uneven fibrosis-stage distribution and may limit the generalizability of stage-specific estimates. In particular, the F1 subgroup included only six participants, limiting the statistical precision of estimates for early-stage fibrosis; therefore, findings for this subgroup should be interpreted cautiously and require confirmation in larger cohorts. Second, confirmation method was closely associated with institution, making it difficult to fully separate pathway-related effects from center-specific acquisition differences. The positive coefficient for biopsy should therefore be interpreted as an association rather than a direct biological effect of the procedure. Needle biopsy is inherently subject to sampling error: the small tissue core obtained (typically 1–2 cm in length) may not accurately represent the global distribution of fibrosis across the whole liver [38], and the ROI locations used for CV measurement may not have precisely corresponded to the biopsy sites. This spatial mismatch may have introduced non-directional misclassification and attenuated the observed association between CV and fibrosis stage. In contrast, systematic underestimation of fibrosis stage by needle biopsy could partly explain the higher CV observed in biopsy-confirmed cases at a given stated stage (B = +1.023). Furthermore, despite adjustment for institution and pathology confirmation method, residual confounding by unmeasured factors such as inflammation grade, steatosis, or concomitant medication use cannot be fully excluded and may influence CV values independently of fibrosis. Third, CV measurements in the primary analysis were performed mainly by a single radiologist (Y.R.K.), which may have introduced measurement bias. Although the high intra- and inter-observer agreement supports the reproducibility of the method, some reader dependence remains because ROIs were placed manually. Fourth, 15-min and 20-min HBP images were acquired at different institutions. Because signal intensity on HBP images may continue to increase between 15 and 20 min after Gd-EOB-DTPA administration in patients with preserved hepatic function, timing differences could theoretically introduce systematic differences in CV values. To address this issue, institution was included as a covariate in the additional adjusted multivariable model, in which fibrosis stage remained independently associated with CV (B = 0.468, *p* < 0.001). In addition, the matched comparison between 15-min and 20-min HBP images showed a slightly lower mean CV in the 15-min group, but the difference was not statistically significant (4.28 ± 0.65 vs. 4.56 ± 0.54, *p* = 0.09). However, the absence of a significant difference does not establish equivalence or exclude residual confounding by timing or other center-specific protocol differences. Fifth, CV measurement relied on custom MATLAB-based software with semi-automatic liver segmentation, which requires manual verification of ROI placement; the development of fully automated ROI placement would improve workflow integration and reproducibility in future applications. The software is available upon reasonable request. MRI protocol differences across institutions were also considered; the matched comparisons did not detect significant differences (Section 3.6), and the association between CV and fibrosis stage remained robust in the adjusted and sensitivity analyses (Supplementary Tables S1 and S2). Finally, all three participating institutions were located in the Republic of Korea, and hepatitis B virus infection was the predominant etiology in the study population. Further external validation is needed in populations with different etiologic distributions, such as MASLD-predominant Western cohorts.

## 5. Conclusions

In this prospective multicenter study with pathologically confirmed fibrosis stages, the coefficient of variation derived from Gd-EOB-DTPA-enhanced hepatobiliary phase MR images showed a strong correlation with METAVIR fibrosis stage (Spearman’s ρ = 0.74) and superior diagnostic performance compared with the serum biomarkers APRI and FIB-4 across most fibrosis thresholds (AUROC, 0.92 for ≥F2; 0.88 for ≥F3; and 0.86 for F4). Pathologic fibrosis stage was the factor most strongly associated with CV, and this association persisted after adjustment for institution and pathology confirmation method. Because CV can be derived from routinely acquired HBP images without additional hardware or laboratory tests, it may serve as a practical non-invasive adjunct for liver fibrosis assessment. These findings were obtained in a Korean HBV-predominant multicenter cohort, and external validation in geographically and etiologically diverse populations, particularly those with MASLD or other etiologies, is warranted.

## Figures and Tables

**Table 1:** Clinical Characteristics of the Participants.

	No Fibrosis (F0, *n* = 19)	Fibrosis Patients (F1–F4, *n* = 80)	*p*-Value
**Demographics**			
Mean Age (years)	37.5 ± 17.5	59.8 ± 10.8	<0.001
Male/Female, *n*	12/7	52/28	1.000
**Etiology of Liver Disease, *n* (%)**			
Normal ^+^	17 (89)	—	
Hepatitis B	—	33 (41)	
Hepatitis C	1 (5)	3 (4)	
Coinfection ^++^	—	3 (4)	
Alcohol	—	14 (18)	
Autoimmune	—	12 (15)	
NAFLD	—	11 (14)	
Idiopathic	—	4 (5)	
Toxic	1 (5)	—	
**Liver Fibrosis Stage ^+++^, *n* (%)**			
F0 (no fibrosis)	19 (100)	—	
F1 (portal fibrosis without septa)	—	6 (8)	
F2 (portal fibrosis with few septa)	—	12 (15)	
F3 (numerous septa without cirrhosis)	—	25 (31)	
F4 (cirrhosis)	—	37 (46)	
**Mean CV (%) by Fibrosis Stage ^++++^ [Mean ± SD]**			
F0 (no fibrosis)	3.41 ± 0.27	—	<0.001
F1 (portal fibrosis without septa)	—	3.78 ± 0.35	
F2 (portal fibrosis with few septa)	—	4.11 ± 0.62	
F3 (numerous septa without cirrhosis)	—	4.39 ± 0.69	
F4 (cirrhosis)	—	5.31 ± 1.06	
**Pathologic Confirmation Methods**			
Biopsy	7	40	
Surgery	12	40	
**Serologic Tests**			
Platelet count (×10^3^/μL)	236.9 ± 38.8	168.5 ± 58.4	<0.001
Albumin (g/dL)	4.3 ± 0.3	4.0 ± 0.6	0.029
Bilirubin (mg/dL)	0.9 ± 1.3	1.0 ± 1.4	0.202
PT (INR)	1.0 ± 0.1	1.7 ± 2.6	0.010
AST (IU/L)	42.1 ± 88.8	62.3 ± 133.0	<0.001
ALT (IU/L)	29.1 ± 46.9	46.5 ± 88.5	0.005
ALP (U/L)	85.5 ± 49.4	151.3 ± 133.8	0.007
GGT (IU/L)	46.0 ± 50.8	88.4 ± 97.0	0.014

Data are presented as mean ± standard deviation unless otherwise indicated. NAFLD, nonalcoholic fatty liver disease; PT (INR), prothrombin time (international normalized ratio); AST, aspartate aminotransferase; ALT, alanine aminotransferase; ALP, alkaline phosphatase; GGT, gamma-glutamyltransferase. ^+^ Healthy controls (*n* = 12) and patients with suspected chronic liver disease but an F0 result (*n* = 5) were included. ^++^ Coinfection was defined as simultaneous infection with hepatitis B and hepatitis C. ^+++^ Fibrosis stage was determined according to the METAVIR fibrosis staging system. ^++++^ Differences between the F0 and F1–F4 groups were assessed using the Mann–Whitney U test for continuous variables and the chi-square test or Fisher’s exact test for categorical variables. Differences in mean CV values across fibrosis stage groups (F0–F4) were assessed using Welch’s analysis of variance (ANOVA), with the Games–Howell test for post hoc pairwise comparisons.

**Table 2:** Univariate and Multivariable Linear Regression Analyses of Factors Associated with CV.

	Univariate Analysis			Multivariable Analysis		
	Coefficient (95% CI)	Standard Error	*p* Value	Coefficient (95% CI)	Standard Error	*p* Value
**Platelet count**	−0.007 (−0.010–−0.003)	0.002	<0.001	−0.001 (−0.004–0.002)	0.001	0.42
**Albumin**	−0.749 (−1.105–−0.393)	0.179	<0.001	−0.313 (−0.622–−0.004)	0.156	0.047
**Bilirubin**	0.100 (−0.053–0.253)	0.077	0.20			
**PT (INR)**	−0.032 (−0.123–0.058)	0.046	0.48			
**AST**	0.000 (−0.002–0.002)	0.001	0.93			
**ALT**	0.000 (−0.003–0.003)	0.001	0.96			
**ALP**	0.003 (0.002–0.005)	0.001	<0.001	0.001 (0.000–0.003)	0.001	0.08
**GGT**	0.001 (−0.001–0.004)	0.001	0.22			
**Pathologic fibrosis stage**	0.465 (0.359–0.570)	0.053	<0.001	0.389 (0.267–0.510)	0.061	<0.001

CV, coefficient of variation; CI, confidence interval; PT (INR), prothrombin time (international normalized ratio); AST, aspartate aminotransferase; ALT, alanine aminotransferase; ALP, alkaline phosphatase; GGT, gamma-glutamyltransferase. Results of the additional model adjusted for institution and pathology confirmation method are presented in Supplementary Table S1.

**Table 3:** ROC Analysis of Mean CV, APRI, and FIB-4 for the Diagnosis of Significant Fibrosis (≥F2), Advanced Fibrosis (≥F3), and Cirrhosis (F4).

		Significant Fibrosis (≥F2)	Advanced Fibrosis (≥F3)	Cirrhosis (F4)
**Mean** **CV**	**AUROC (95% CI)**	0.92 (0.86–0.97)	0.88 (0.81–0.95)	0.86 (0.79–0.94)
	**Optimal cutoff**	3.84	4.01	4.50
	**Sensitivity**	82.4	87.1	81.1
	**Specificity**	96.0	78.4	83.9
	***p *** **value**	<0.001	<0.001	<0.001
	Comparison with APRI *	0.045	0.005	<0.001
	Comparison with FIB-4 *	0.09	0.03	0.006
**APRI**	**AUROC (95% CI)**	0.80 (0.70–0.91)	0.71 (0.60–0.83)	0.68 (0.57–0.79)
	**Optimal cutoff**	0.32	0.32	0.41
	**Sensitivity**	89.2	90.3	83.8
	**Specificity**	68.0	51.4	46.8
	***p *** **value**	<0.001	<0.001	0.001
**FIB-4**	**AUROC (95% CI)**	0.82 (0.71–0.93)	0.75 (0.65–0.86)	0.71 (0.60–0.81)
	**Optimal cutoff**	1.12	1.12	1.44
	**Sensitivity**	94.6	96.8	91.9
	**Specificity**	64.0	48.6	38.7
	***p *** **value**	<0.001	<0.001	<0.001

ROC, receiver operating characteristic; AUROC, area under the receiver operating characteristic curve; CV, coefficient of variation; APRI, aspartate aminotransferase-to-platelet ratio index; FIB-4, fibrosis-4 index. * *p* values for comparisons of AUROCs are shown.

## Data Availability

The datasets used and/or analyzed during the current study are available from the corresponding author upon reasonable request; restrictions apply because of participant privacy and institutional review board requirements. The CV analysis software is also available from the corresponding author upon reasonable request.

## Supplementary Materials

The following supporting information can be downloaded at: https://www.mdpi.com/article/10.3390/jcm15207996/s1, Supplementary Methods: Imaging Acquisition and CV Analysis; Table S1: Multivariable linear regression model for CV in the full cohort (*n* = 99) with adjustment for institution and pathology confirmation method; Table S2: Sensitivity analysis: multivariable linear regression model for CV restricted to surgically confirmed cases (*n* = 52).
