# Supplementary material for: Assessment of Liver Fibrosis Using the Coefficient of Variation on Gadoxetic Acid-Enhanced Hepatobiliary Phase MR Imaging: A Prospective Multicenter Study
Source: J Clin Med. 2026 Oct 15;15(20):7996. doi: 10.3390/jcm15207996 (PMC13706113; doi:10.3390/jcm15207996)
Supplement: Supplementary file 1 [file jcm-15-07996-s001.zip › jcm-4575517-supplementary.pdf]

Supplementary Materials

Supplementary Material for: "Assessment of Liver Fibrosis Using the Coefficient of Variation on Gadoxetic Acid-Enhanced Hepatobiliary Phase MR Imaging: A Prospective Multicenter Study"

Kim YR, Kim TH, Jeong CW, Lee YH, Cho EY, Choi KH, Jeong YY, Heo SH, Kim KW, Ko Y, Shin Y, Kang DM, Yoon KH

Journal of Clinical Medicine | Section: Gastroenterology & Hepatology

Imaging Acquisition

1) Institution A obtained 20-min HBP images using two 3.0-Tesla (T) scanners. Gd-EOB-DTPA was injected at 0.1 mL/kg body weight at a rate of 1.5 mL/s using a power injector, followed by a 15-mL saline flush. ① n = 48 (Ingenia CX, Philips Healthcare): T1-weighted images were acquired using a three-dimensional T1 eTHRIVE pulse sequence: TR/TE = 3.17/1.57 msec; FOV = 38 × 38 cm; matrix, 320 × 234; slice thickness, 2.5 mm; NEX = 1; scan time = 9 sec. ② n = 2 (Achieva, Philips Healthcare): T1-weighted images were acquired using a three-dimensional T1 eTHRIVE pulse sequence: TR/TE = 3.15/1.55 msec; FOV = 37 × 37 cm; matrix, 312 × 203; slice thickness, 2.5 mm; NEX = 1; scan time = 9 sec.

2) Institution B obtained 15-min HBP images using a single 3.0-T scanner. Gd-EOB-DTPA was injected at 0.1 mL/kg body weight at a rate of 1 mL/s, followed by a 20-mL saline chaser. n = 29 (Magnetom Skyra, Siemens Healthcare): T1-weighted images were acquired using a three-dimensional T1 VIBE pulse sequence: TR/TE = 4.0/1.95 msec; FOV = 38 × 28 cm; matrix, 384 × 218; slice thickness, 3.0 mm; NEX = 1; scan time = 9 sec.

3) Institution C obtained 20-min HBP images using two different 3.0-T scanners. Gd-EOB-DTPA was injected at 0.1 mL/kg body weight at a rate of 1.0 mL/s using a power injector, followed by a 20-mL saline flush. ① n = 19 (Magnetom Skyra, Siemens Healthcare): T1-weighted images were acquired using a three-dimensional T1 VIBE pulse sequence: TR/TE = 3.39/1.33 msec; FOV = 38 × 24 cm;

matrix, 384 × 240; slice thickness, 3.0 mm; NEX = 1; scan time = 9 sec. ② n = 1 (Ingenia CX, Philips Healthcare): T1-weighted images were acquired using a three-dimensional T1 mDIXON pulse sequence: TR/TE = 4.1/1.33 msec; FOV = 35 × 35 cm; matrix, 320 × 256; slice thickness, 3.0 mm; NEX = 1; scan time = 9 sec.

CV Analysis

To correct for signal intensity inhomogeneity in abdominal MR images, we adopted a simple multiplicative–additive model of intensity inhomogeneity. On the basis of the physics of MR imaging, an acquired image (I) can be expressed as follows:

$$I = bJ + n \qquad (1)$$

where b represents the field bias, J is the true image, and n is additive noise. The true image J is assumed to be piecewise constant because it reflects the intrinsic physical properties of the imaged object. The field bias b is assumed to vary slowly across the image, and the additive noise n is assumed to be zero-mean Gaussian noise.

In this study, the acquired image I was regarded as a function defined in the continuous domain, as in previous studies (references 22 and 23 in the main text). This model describes images in which signal intensity inhomogeneity arises from the bias field component (b).

CV was calculated within each ROI using the following equation:

CV (%) = (standard deviation of pixel signal intensities within the ROI / mean pixel signal intensity within the ROI) × 100 (2)

Supplementary Tables

Table S1. Multivariable linear regression model for CV in the full cohort (n = 99) with adjustment for institution and pathology confirmation method. Fibrosis stage remained independently associated with CV (B = 0.468, P < 0.001) after adjustment for albumin, institution, and pathology confirmation method. All variance inflation factors (VIFs) were below 5.

Coding: institution (1 = Institution A, 2 = Institution B, 3 = Institution C); pathology confirmation method (0 = surgery, 1 = biopsy).

Variable	B	SE	t	P	95% CI	VIF
Intercept	3.541	0.799	4.430	<0.001	1.953 to 5.128	
Fibrosis stage (0–4)	0.468	0.055	8.468	<0.001	0.358 to 0.578	1.307
Albumin	−0.341	0.138	−2.473	0.015	−0.615 to −0.067	1.115
Institution	0.366	0.188	1.945	0.055	−0.008 to 0.740	4.204
Biopsy (vs. surgery)	1.023	0.287	3.560	0.001	0.452 to 1.593	3.950

Table S2. Sensitivity analysis: multivariable linear regression model for CV restricted to surgically confirmed cases (n = 52). Fibrosis stage remained independently associated with CV (B = 0.381, P < 0.001) in surgically confirmed cases.

Coding: institution (0 = Institutions A/B, 1 = Institution C).

Variable	B	SE	t	P	95% CI	VIF
Intercept	4.655	0.985	4.727	<0.001	2.675 to 6.635	
Fibrosis stage (0–4)	0.381	0.082	4.654	<0.001	0.216 to 0.546	1.793
Albumin	−0.393	0.202	−1.944	0.058	−0.800 to 0.013	1.244
Institution	0.447	0.241	1.857	0.069	−0.037 to 0.932	1.578
